# Social support and strain and emotional distress among Latinos in the northeastern United States

**DOI:** 10.1186/s40359-021-00544-3

**Published:** 2021-03-07

**Authors:** Shir Lerman Ginzburg, Stephenie C. Lemon, Eric Romo, Milagros Rosal

**Affiliations:** 1 Department of Public Health Sciences, UConn Health, 263 Farmington Ave, Farmington, CT 06032 USA; 2 Division of Preventive and Behavioral Medicine, University of Massachusetts Medical School, Worcester, MA 01655 USA; 3 Department of Clinical and Population Health, University of Massachusetts Medical School, Worcester, MA 01655 USA

**Keywords:** Ataque de Nervios, Emotional distress, Latinos, Social networks, Social support, Social strain

## Abstract

**Background:**

US Latinos report high levels of emotional distress. Having positive familial and friend social support buffers emotional distress among US Latinos, but thus far no research has been done on social support and ataque de nervios in that population, or on social strain and emotional distress.

**Methods:**

This paper assesses social support and strain across three relationship types (partner, family, and friends) with three measures of emotional distress (depression, anxiety, and ataque de nervios). The sample for partner, family, and friend support included 508 Latino adults 21 and older. Multivariate logistic regression models were used to assess the association of social support and strain with each outcome.

**Results:**

As all social support types increased, the odds of emotional distress symptoms decreased. Conversely, as each unit of partner and family strain increased, the odds of emotional distress symptoms increased. Increased friend strain was associated with greater odds of depressive and anxiety symptoms only.

**Conclusion:**

Social support in all three network types (partner, family, and friend) was associated with a decrease in the odds of emotional distress, assessed as symptoms of depression, anxiety, and ataque de nervios.

## Introduction

Latinos in the United States are at heightened risk for emotional distress, compared to non-Latino whites [1–3]. Emotional distress is a broad category of mental suffering that encompasses symptoms such as anxiety, depression, and physical symptoms such as fatigue, body aches, and appetite changes [1, 3]. Previous studies suggest that as many as 27% of Latinos experience elevated depressive symptoms any given time [4], and 11% experience elevated symptoms of anxiety [5]. By comparison, 7.9% of Non-Hispanic Whites (NHW) experience elevated depressive symptoms [6, 7], and 9% suffer from anxiety [8, 9]. Furthermore, 14% of Latinos report a lifetime history of ataque de nervios [10], a cultural syndrome which the Diagnostic and Statistical Manual (DSM)-5 [11] classifies as a culturally-specific category of aberrant behaviors that don’t fit any other DSM disorders [12]. A central feature of an ataque de nervios is a sense of being out of control; common symptoms include uncontrollable shouting, and attacks of crying. Attacks are often triggered by a stressful event related to the family [13]. While ataque de nervios is a distinct cultural syndrome, it is strongly associated with depression and anxiety disorders [10, 14]. However, unlike depression or anxiety episodes, which tend to be generalized and prolonged conditions, episodes of ataque de nervios are shorter and occur in response to specific, socially-accepted triggers [15, 16]. Ataque de nervios is a marker of social vulnerability, or the structural factors (such as poverty, discrimination, and immigration status) that adversely affect certain populations and which are themselves associated with emotional distress [14, 17, 18]. In Latino populations, ataque de nervios is a marker for both the aforementioned structural factors and for interpersonal conflict (such as social strain), and is a culturally acceptable way of expressing distress [14, 17, 19]. Expressing ataque de nervios might therefore be indicative of socioeconomic and interpersonal vulnerabilities that necessitate assistance. Given that Latinos constitute the largest ethnic minority group in the US (18.5% of the United States population as of 2019) [20], understanding the root causes of mental health disparities and addressing mental health needs in this population is of critical importance.

Social Convoy Theory posits that individuals are surrounded by both familial and non-familial networks that provide for exchange of social support which protects against emotional distress and protects well-being [21, 22]. Previous research supports Social Convoy Theory by showing that social support from partners and family is associated with decreased depressive and anxiety symptoms, positive affect, and life satisfaction among adults [23–27]. In the National Latino and Asian American Survey (NLAAS)’s data of 2554 Latinos, [28] examined sources of Latino social support including family and friends, and found that having family support was associated with lower scores on depressive and anxiety symptomology, and friend support had a smaller but still meaningful reduction on depressive symptomology. Merz et al. [29] found that social support of any kind was inversely related with hyptertension among Latinos, while Crockett et al. [30] found that having a strong familial social support network led to better emotional adjustment for Latino college students and protected them against external stressors.

Conversely, social strain, or the experience of negative social interactions, can negatively impact an individual’s mental health [21, 26, 31]. Grzywacz et al. [32] found that partner strain was associated with increased levels of emotional distress among immigrant Mexican women, and Buchanan and Smokowski [33] found that family and friend strain were both associated with emotional distress among Latino adolescents. Social strain has been found to increase the risk of anxiety and depression among other collectively-oriented populations, such as Koreans, since social strain indicates a break in highly-prized interpersonal relationships [34, 35]. More recent literature on emotional distress and social relatioships among Latino social networks have not thoroughly addressed social strain. Furthermore, relatively little research has examined the association of social support and strain and ataque de nervios among Latino populations [19, 36].

Social support and strain may be particularly important for the emotional well-being of Latinos due to the strong emphasis Latino place on close social support networks [23, 34, 37]. In particular, family, friend, and partner networks are highly valued, more so than other networks (like colleagues), due to the closeness of family (biological and partnership) relationships and the emphasis on family and friend loyalty [28, 36, 38]. Given the centrality of interpersonal relationships in Latino cultures [13, 28, 38] and the high rates of emotional distress among Latinos in the US [4, 5, 36, 39], further examination of the associations between social support and strain and emotional distress, particularly ataque de nervios, is warranted.

The purpose of this paper is to assess the association between two dimensions of social relationships, namely social support and social strain across three relationship types (partner, family, friends), with several measures of emotional distress, including symptoms of depression, anxiety, and ataque de nervios symptomology outcomes in a sample of Latinos residing in the northeastern United States. We hypothesized that social support from partners, family, and friends would be protective against risk for emotional distress, while social strain from partners, family, and friends was hypothesized to increase the risk of emotional distress.

## Methods

### Study design

This is a cross-sectional study using data from the Lawrence Health and Well Being (LHWB) Study. The primary goal of the LHWB Study was to understand factors associated with mental health care utilization of low-income Latinos residing in the northeastern United States. Patients were recruited from a large community health center in Lawrence, MA, which has a majority (73%) Latino population [40]. Inclusion criteria included being of Latino/Hispanic ethnicity, Spanish or English speaking, between 21 and 85 years of age, no plans to move out of the area within the study period (one year), no cognitive impairments precluding participation (i.e. answering verbally administered questions), and able and willing to give informed consent. Patients were excluded if they had cognitive impairments, a psychiatric hospitalization within the past six months, or planned to move out of the area within the study period.

Proportional sampling was used to randomly select patients from electronic health records from six predefined age by gender strata. Randomly selected participants were mailed invitation letters in English and Spanish signed by the chief medical officer that described the study, informed patients they would be contacted by phone, and provided a toll-free number to call if they did not wish to participate. Within two weeks of the mailing, bilingual/bicultural coordinators from the local community contacted patients over the phone to describe the study and ask questions, screen for eligibility, and invite eligible individuals to participate. Individuals who were eligible and interested were scheduled for a study assessment visit at a central location in the community. Written informed consent was obtained by the community coordinator and study surveys were administered verbally. This study was approved by the Institutional Review Board at the University of Massachusetts Medical School.

### Sample

This study uses three analytic samples, one for each relationship type under evaluation (family, partner, or friend). The entire Lawrence Health and Well Being (LHWB) Study includes 602 respondents, although each set of analyses were limited to individuals who reported having the respective relationship type (i.e., partner, family, friends). The sample for analyses involving family support and strain included 593 participants and was thus used for descriptive analysis. Participants were excluded from the family sample if they were missing complete data to calculate a family support or strain score (n = 4) or were missing data on a potential confounder of interest (n = 5). The final family sample included 593 participants.

The sample for analyses assessing partner support and strain excluded two hundred thirty-four participants who reported not currently having a spouse or partner. Other participants were excluded from the partner sample if they were missing complete data to create a partner support or strain score (n = 9) or were missing data on a potential confounder of interest (n = 1). The final partner sample included 358 participants.

The sample for analyses involving friend support and strain included 508 participants, excluding eighty-one participants who reported “Never or hardly ever” being in contact with any of their friends; therefore, they did not complete the survey measures for friend support or strain. Other participants were excluded from the friend sample if they were missing complete data to calculate a friend support or strain score (n = 8) or were missing data on a potential confounder of interest (n = 5). The final friend sample included 508 participants.

### Measures

#### Emotional distress measures

We evaluated three emotional distress symptoms: depressive symptoms, anxiety symptoms, and *ataque de nervios* symptoms.

#### Depressive symptoms

Depressive symptoms were assessed using the Centers for Epidemiologic Studies—Depression scale (CES-D). This 20-item survey measure assesses the frequency of various depressive symptoms in the past week on a 4-point scale ranging from “Less than 1 day (rarely or none of the time)” to “5–7 days (most or all of the time)”. The total CES-D score was calculated as a sum of the responses to all 20 questions, with a higher score indicating the presence of more depressive symptoms. A score of 16 or greater is indicative of a clinically significant level of depressive symptoms. Therefore, the outcome of depressive symptoms was treated as a dichotomous variable, with a score ≥ 16 indicating clinically significant depressive symptoms and a score < 16 indicating symptoms with no clinical significance. The CES-D was previously translated and adapted for interviewer administration, worth good internal reliability (alpha = 0.87) and adequate test–retest reliability among Latinos [41].

#### Anxiety symptoms

Anxiety symptoms were assessed with the Generalized Anxiety Disorders scale (GAD-7). This 7-item survey assesses the frequency of various symptoms in the past two weeks on a 4-point scale. Possible response options are “not at all”, several days”, “more than half the days” and “nearly every day”. Participants were coded as having elevated anxiety symptoms if they scored ≥ 10, a cut-off which is valid for Latinos in the U.S. and is valid for both Spanish and English versions of the scale after measuring for internal reliability using Cronbach’s alpha (Mills et al. 2014). The outcome of anxiety symptoms was treated as a dichotomous variable, with a score ≥ 10 indicating elevated anxiety symptoms and a score < 10 indicating symptoms with no clinical significance.

#### Ataque de nervios

Ataque de nervios is a cultural syndrome of emotional distress among Latinos in which individuals reported experiencing at least 4 symptoms on the NLAAS’s ataque de nervios questionnaire. Ataque de nervios was assessed using the questionnaire developed by the National Latino and Asian American Survey [14]. This 16-question survey is broken down into two parts. The first part asks the participant if he or she has ever had an ataque de nervios. If not, the questionnaire ends there. If yes, the survey asks if the participant experienced any of the following 14 symptoms: anger or rage, aggression, hysteria, dizziness, seizures, heart palpitations, chest tightness, fainting, shouting a lot, having crying attacks, feeling very scared or frightened, having a period of amnesia, and trembling. Finally, the participant is asked if he or she experienced an ataque de nervios in the past 2 weeks. Participants were coded as having experienced ataque de nervios if they reported having had an episode of ataque de nervios and also reported experiencing at least four of the fourteen symptoms [14]. The outcome of ataque de nervios symptoms was treated as a dichotomous variable, with a score ≥ 4 indicating elevated ataque de nervios symptoms and a score < 4 indicating symptoms with no clinical significance.

#### Social Support and Strain

Three different sources of social support and strain were assessed: partners, family, and friends. The partner support scale included two additional questions: How much does he or she appreciate you? How much can you relax and be yourself around him or her? All six survey measures (partner, family, and friend support and strain) were taken from [27] study on social support and strain indicators. Walen and Lachman had significant Cronbach Alpha scores for each scale: family support (0.82), family strain (0.80), friend support (0.88), friend strain (0.79), partner support (0.86) and partner strain (0.81). Family and friend support included 4-items each and partner support was comprised of 6-items. All items used a 4-point Likert scale ranging from 1 = a lot; 4 = not at all: (i) How much do [members of your family, your spouse or partner, or your friends] really care about you? (ii) How much do they understand the way you feel about things? (iii) How much can you rely on them if you have a serious problem? (iv) How much can you open up to them if you need to talk about your worries?

Similarly, family and friend strain were measured using 4-item scales, and partner strain was measured using a 6-item scale. The following 4 items were common to all three scales (1 = often, 4 = never): (i) How often do [members of your family, your spouse or partner, or your friends] make too many demands on you? (ii) How often do they criticize you? (iii) How often do they let you down when you are counting on them? (iv) How often do they get on your nerves? the partner strain scale included two additional questions: How often does he or she make you tense? How often does he or she argue with you?

An individual’s score for each measure was calculated by summing the items and taking the average. All support and strain scores were therefore treated as continuous variables, with possible scores ranging from 1 to 4. Items were recoded such that a higher score indicated higher support or strain.

#### Covariates

Covariates assessed in the present study included self-reported age, sex, education, place of birth, perceived income, household size, and caregiving status. Age was used categorically, consistent with the strata used for study recruitment purposes (21–34, 35–54, ≥ 55 years) and with research showing that social support networks shrink as individuals enter old age^18^. The highest level of education was used and categorized as “less than high school”, “high school graduate” and “some college or college graduate”. Place of birth included the Dominican Republic, Puerto Rico, the mainland United States and ‘other’. Perceived income was measured with a single item that asked “In general, would you say you (and your family living in the same household) have more money than you need, just enough money for your needs, or not enough money to meet your needs?”. Response options were collapsed to “not enough” and “just enough or more than enough” due to low responses in the “more than enough” category. This measure of perceived income was selected over annual household income because this measure is prone to missingness.

### Statistical analysis

Frequencies distributions and means with standard deviations were used to describe the study sample. Separate logistic regression models estimated the associations between each support and strain variable (partner, family, friend) and each emotional distress outcome (depressive, anxiety, and *ataque* symptoms). Crude models were first computed. Multivariable models were then developed. We used the 10% technique to determine which covariates changed the crude odds ratio results by at least 10%. None of the covariates changed the crude odds ratio by more than 4% in any of the models. Therefore, only basic sociodemographic covariates (age, sex, place of birth, perceived income, education) were included in the multivariable models. Statistical significance was set at *p* < 0.05. STATA version 14 was used for all analyses [42].

The Institutional Review Board of the University of Massachusetts Medical School approved the parent study from which these data were taken. Since this paper consists of retrospective data analysis, no IRB approval was required for this paper’s data analyses.

### Results

In the sample used for all support/strain analysis, 51.16% of participants were female, 41.86% had a high school degree or less, and the mean age was 46.6 years (SD = 0.63). Demographic characteristics, stratified by emotional distress outcome, are presented in Table 1. More than one third (36.2%) of the total sample had elevated depression symptoms, 21.9% had elevated symptoms of anxiety and 27.3% met criteria for ataque de nervios. The mean partner support score is 3.60, the mean family support score is 3.44, and the mean friend support score is 3.20 (all out of a potential score of 4). The mean partner strain score is 3.06, the mean family strain score is 3.34, and the mean friend strain score is 3.01 (also all out of a potential score of 4).

**Table 1:** Characteristics of the Study Sample by Mental Health Outcome Status in the Latino Health and Well-Being Study, N = 593

	Depressive Symptoms		Anxiety Symptoms		Ataque de Nervios	
	*Yes* *n* = *253*	*No* *n* = *340*	*Yes* *n* = *130*	*No* *n* = *463*	*Yes* *n* = *162*	*No* *n* = *431*
	*n (%)*	*n (%)*	*n (%)*	*n (%)*	*n (%)*	*n (%)*
*Age group*						
21–34	75 (29.6%)	106 (31.2%)	41 (31.5%)	140 (30.2%)	53 (32.7%)	128 (29.7%)
35–54	96 (37.9%)	109 (32.1%)	48 (36.9%)	157 (33.9%)	62 (38.3%)	143 (33.2%)
55 +	82 (32.4%)	125 (36.8%)	41 (31.5%)	166 (35.9%)	47 (29%)	160 (37.1%)
*Gender*						
Male	100 (39.5%)	189 (55.6%)	54 (41.5%)	235 (50.8%)	68 (42%)	221 (51.3%)
Female	153 (60.5%)	151 (44.4%)	76 (58.5%)	228 (49.2%)	94 (58%)	210 (48.7%)
*Place of Birth*						
Dominican Republic	160 (63.2%)	254 (74.7%)	76 (58.5%)	338 (73%)	102 (63%)	312 (72.4%)
Puerto Rico	47 (18.6%)	38 (11.2%)	21 (16.2%)	64 (13.8%)	33 (20.4%)	52 (12.1%)
Continental U.S	29 (11.5%)	17 (5%)	22 (16.9%)	24 (5.2%)	19 (11.7%)	27 (6.3%)
Other	17 (6.7%)	31 (9.1%)	11 (8.5%)	37 (8%)	8 (4.9%)	40 (9.3%)
*Perceived income*						
Not enough money	159 (62.8%)	129 (37.9%)	94 (72.3%)	194 (41.9%)	89 (54.9%)	199 (46.2%)
Just enough/more than enough	94 (37.2%)	211 (62.1%)	36 (27.7%)	269 (58.1%)	73 (45.1%)	232 (53.8%)
*Education level*						
< High school	109 (43.1%)	135 (39.7%)	54 (41.5%)	190 (41%)	67 (41.4%)	177 (41.1%)
High school graduate	79 (31.2%)	96 (28.2%)	45 (34.6%)	130 (28.1%)	49 (30.2%)	126 (29.2%)
Some college/college graduate	65 (25.7%)	109 (32.1%)	31 (23.8%)	143 (30.9%)	46 (28.4%)	128 (29.7%)

Crude and multivariate models are presented in Table 2. Statistically significant relationships are bolded. In multivariable models, each unit increase in family support score was associated with a 43% decrease in depressive symptoms (OR = 0.57, 95% CI = 0.45–0.72), a 41% decrease in anxiety symptoms (OR = 0.59, 95% CI = 0.46–0.75), and 16% decrease in symptoms of ataque de nervios (OR = 0.84, 95% CI 0.67–1.07). Conversely, each unit increase in family strain score was associated with more than twice the odds of depressive symptoms (OR = 2.22, 95% CI = 1.69–2.90) and anxiety symptoms (OR = 2.67, 95% CI 1.99–3.60) and a 60% increase in having experienced ataque de nervios (OR = 1.60, 95% CI = 1.24–2.06).

**Table 2:** Adjusted and Unadjusted odds ratios of the relationships between family (n = 593), partner (n = 358) and friend (n = 508) support and strain with depressive symptoms, anxiety symptoms and *ataque de nervios* among participants in the Latino Health and Well-being Study

	Depressive Symptoms		Anxiety Symptoms		Ataque de Nervios	
	Unadjusted	Adjusted	Unadjusted	Adjusted	Unadjusted	Adjusted
	(95% CI)	(95% CI)	(95% CI)	(95% CI)	(95% CI)	(95% CI)
Family Support	0.55	0.57	0.55	0.59	0.79	0.84
	(0.44–0.69)	(0.45–0.72)	(0.44–0.70)	(0.46–0.75)	(0.63–0.99)	(0.67–1.07)
Family Strain	2.04	2.22	2.38	2.67	1.66	1.6
	(1.62–2.58)	(1.69–2.90)	(1.85–3.07)	(1.99–3.60)	(1.32–2.10)	(1.24–2.06)
Partner Support	0.29	0.31	0.37	0.4	0.41	0.41
	(0.19–0.49)	(0.20–0.49)	(0.25–0.53)	(0.27–0.60)	(0.28–0.59)	(0.28–0.61)
Partner Strain	2.46	2.58	2.62	2.75	2.14	2.3
	(1.84–3.29)	(1.87–3.57)	(1.88–3.67)	(1.88–4.02)	(1.60–2.86)	(1.67–3.17)
Friend Support	0.71	0.69	0.78	0.78	0.86	0.87
	(0.58–0.88)	(0.55–0.86)	(0.61–0.99)	(0.60–1.01)	(0.69–1.08)	(0.69–1.09)
Friend Strain	1.44	1.62	1.51	1.53	1.42	1.42
	(1.08–1.93)	(1.17–2.26)	(1.09–2.09)	(1.07–2.19)	(1.04–1.93)	(1.02–1.96)

An increase in partner support was associated with a significant decrease in depressive symptoms, anxiety symptoms and ataque de nervios (OR = 0.31, 95% CI 0.20–0.49; OR = 0.40, 95% CI 0.27–0.60; OR = 0.41, 95% CI 0.28–0.61, respectively). Conversely, an increase in partner strain was associated with a significant increase in depressive symptoms, anxiety symptoms and ataque de nervios (OR = 2.58, 95% CI 1.87–3.57; OR = 2.75, 95% CI 1.88–4.02; OR = 2.30, 95% CI 1.67–3.17, respectively).

Friend support was significantly associated with just one of our outcomes. A one-unit increase in friend support was associated with a 31% decrease in depressive symptoms (OR = 0.69, 95% CI 0.55–0.86). That trend was observed for anxiety symptoms and ataque de nervios, but the results did not reach statistical significance. Friend strain resulted in an increase in all three psychological symptoms, but none of the relationships reached statistical significance.

### Discussion

In this study, we found that social support in all three network types (partner, family, and friend) was associated with a decrease in the odds of emotional distress, assessed as symptoms of depression, anxiety, and ataque de nervios. Conversely, as partner, family, and friend strain increased, so did the odds of depressive, anxiety, and ataque de nervios symptoms. Our results confirm prior literature that social support from partners and friends reduces risk of emotional distress among Latinos, whereas strained relationships with family, partner and friends are associated with greater odds of experiencing elevated emotional distress. Other studies among non-Latino populations found similar results. For example, Sangalang and Gee [35] found that family support was associated with decreased odds of depressive and anxiety symptoms among Asian Americans participating in NLAAS. However, the same study found that friend strain was only associated with increased depressive symptoms among women, but not men. Roth et al. [43] used the CES-D to measure depressive symptoms among African-American and White familial caregivers who experienced support and strain, and found that among both African-American and White caregivers, those who experienced strain at work also experienced higher rates of depressive symptoms than did caregivers who did not experience strain caring for family (33.92% of high-strain caregivers reported significant CES-D scores compared to 12.54% of moderate-strain caregivers and 8.93% of low-strain caregivers). However, Roth and colleagues did not measure partner and friend social support and strain. Kutschke et al. [44] used the same social support and strain measures as this study to assess partner, family, and friend social support and strain among twins in the US, with women reporting more support from family and friends, while men reported more support from partners. Converseley, both women and men reported similar levels of partner and friend strain, and women reported higher levels of friend strains. Women reported higher levels of both support and strain from their twins than did men. However, Kutschke and colleagues did not measure emotional distress in this population. Teo et al. [45] also used the same social support and strain measures as this study to assess the association between depressive symptomology and partner, family, and friend social support and strain, using the Composite International Diagnostic Interview Short Form (CIDI-SF) to measure depressive symptomology. However, Teo and colleagues did not measure anxiety or ataque de nervios. To our knowledge, no other study has measured the association of ataque de nervios and social support and strain in any population.

Partner support and strain had the strongest associations with each of the three emotional distress indicators out of the three studied sources of social supportand strain, suggesting that partners are highly valued in this population. Our results showing that partner support is associated with depressive symptomology confirms prior literature [4], and our results extend this literature to anxiety and ataque de nervios symptoms. Additionally, our findings extend the literature indicating the negative impact that partner strain can have on emotional states in this population. Given the effort often dedicated to maintaining partner relationships, the diminished self-worth and devalued identity following the failure of a romantic relationship [39], and the mutual commitments (e.g., children, acquaintances, and material possessions) that partners share, partner strain among adults of all ages can potentially exacerbate negative mental health outcomes compared to the other two relationship types.^1^

Previous studies with Latino populations have suggested that family support may offer coping mechanisms against life’s difficulties, buffering the risk of emotional distress [46, 47], while family and partner strain may exacerbate life stressors [39, 48]. Familial support may furthermore provides a safe space for individuals to discuss concerns and problems, particularly following disturbing events [48–50]. Family support have been shown to protect against risk-taking behaviors such as substance abuse and underage unprotected sex, and decreases overall recovery time [28, 48]. Our results extend this literature by specifically examining emotional distress outcomes. Given the centrality of the family in Latino cultures [34, 46–48], it makes sense that positive family support may positively impact emotional health outcomes and negative familial relationships may increase the risk of increased depressive, anxiety, and ataque de nervios symptomology.

Conversely, fraying familial relationships can exacerbate the impact of adverse life circumstances; for example, [51] found that spousal conflict mediate the relationship between poverty and children’s emotional distress and exacerbates familial strain. However, the literature on family strain among Latinos is limited and raises questions on what contributes to family strain in this population, particularly since family strain was reported to be moderately high (3.34/4.00). The emphasis on family in Latino cultures underscores the need for research on the impact of negative family relationships on emotional distress in this population.

This study has strengths and weaknesses. Strengths include the inclusion of ataque de nervios as a mental health outcome, the inclusion of friend support and strain, and the focus on adults, rather than on adolescents or college students. Limitations include the cross-sectional design, which does not allow the assessment of causality. Since this study is cross-sectional, further research is needed to determine whether partners, in addition to having a significant role within the familial sphere, could be instrumental in serving as additional support for individuals with negative mental health outcomes and could mitigate negative mental health symptoms. An additional limitation is that this study did not address Latino subgroup differences. Further research is needed to measure differences in mental health outcomes between different Latino subgroups (e.g., Dominicans, Mexicans) in order to tailor treatment to specific needs. Additionally, this study did not allow the assessment of the association between social support and strain on seeking mental health treatment, which is also an important area for future research on the potential utilization of different support networks for mental health treatment. In addition, since we limited each set of analyses to individuals who reported having the respective relationship type (e.g., friends), this approach might bias the results. For example, having no partner might impact the value and potential impact of another relationship (i.e., family or friends) as a source of support, particularly if the exclusion of those who reported not having the relationship in question may mean that those with the lowest support or greatest strain may have been excluded if the reason for not having the relationship is that they were experiencing low support or high strain. Furthermore, a high number of study participants (n = 82, or 14% of the original sample) did not have social support from friends or may not have had friends due to strained friendships, which might have affected the friendship strain results. We are furthermore unable to differentiate between types of support, such as emotional vs. financial support.

These data on the associations of emotional distress and social support and strain among Latinos, are critical for public health research in that population. This paper emphasizes the importance of social support, particularly family and partner support, among Latinos with emotional distress. Given that Latinos are the fastest growing minority in the United States [52], research on emotional distress and social support and strain among Latinos are critical for understanding mental health in a growing subset of the U.S. population [28, 29, 49]. The results on social strain and emotional distress highlight the critical need for further research on this topic, especially as the variables that trigger social strain are currently not well-understood.

## Conclusions

The paper highlights the importance of interpersonal relationships and their potential associations with emotional distress. We observed that greater social support from partners was associated with decreased likelihood of elevated depression, anxiety and ataque de nervios symptoms; increased support from family support with depression and anxiety symptoms and increased support from friends with depression symptoms only. We also found that the experience of social strain from partners, families, and friends were associated with increased likelihood of elevated depression, anxiety, and ataque de nervios symptoms.

A large proportion of Latinos in this study reported high levels of emotional distress as evidenced by scores on measures of depressive, anxiety and ataque de nervios symptoms. Our study’s inclusion of friends as sources of support and strain is uncommon among studies of Latino populations. Our findings do confirm Mulvaney-Day and colleagues (2007)’s hypothesis that friend support networks, in addition to family, have positive effects on depression symptoms among Latinos. However, associations between friend support with anxiety and ataque de nervios were not observed in this study. Having friends has been shown to increase the sense of belonging; friendship could serve as a protective factor against negative mental health symptoms [28, 49, 50] also found that social support mitigates the effects of emotional distress among low-income communities, particularly those that do not offer many alternative sources of companionship. Social strain might translate into the withdrawal of the comforts that social support provides, such as a sense of community belonging and protection against the unknown, but no data are available on the mechanisms by which social support impacts emotional distress, and further research on this topic is needed [24, 29, 53]. Our research adds to these studies by finding that strained partner, family, and friend relationships negatively impact emotional distress outcomes. Particularly, friend strain had a more consistent association across types of emotional distress outcomes than did friend support, which suggests that more research needs to be done on the association of friend support and strain, and emotional distress outcomes.

This paper is unique in its examination of social strain in relationship to indicators of emotional distress, and in the inclusion of ataque de nervios as an indicator of emotional distress. While only partner support was associated with lower likelihood of ataque de nervios, strain from each of the three relationship types examined was associated with greater likelihood of distress. Prior studies in non-Latino populations have identified social factors associated with ataque de nervios including divorce and experiencing long-standing disagreements with family members [10, 14, 19, 54]. Our findings confirm that family strain increase the risk for experiencing ataque de nervios among Latinos. Our findings that friend strain was also associated with having an ataque de nervios is unique. These data suggest that friend strain is sufficiently significant in this population, and thus social conflict outside of the family may impact emotional distress. This suggests the need for further research on the importance of friend strain in this population.

## Data Availability

The datasets used and/or analysed during the current study are available from the corresponding author on reasonable request.

## Abbreviations

CES-D: Centers for Epidemiologic Studies Depression scale
CIDI-SF: Composite International Diagnostic Interview Short Form
DSM: Diagnostic and Statistical Manual
GAD-7: Generalized Anxiety Disorders Scale
NLAAS: National Latino and Asian American Survey
SAMHSA: Substance Abuse and Mental Health Services Administration
STATA: Software for Statistics and Data Science

## Footnotes

1 Given the cross-sectional nature of these data, this is not something we can determine.
